# THE ENDOPLASMIC RETICULUM PROTEIN QUALITY CONTROL ADAPTATION IN A LONG-LIVED C. ELEGANS PROTEASOMAL MUTANT

**DOI:** 10.1093/geroni/igz038.373

**Published:** 2019-11-08

**Authors:** Meghna N Chinchankar, Karl Rodriguez, Alfred Fisher

**Affiliations:** 1 UT Health San Antonio, San Antonio, Texas, United States; 2 University of Nebraska Medical Center, Omaha, Nebraska, United States

## Abstract

Protein degradation mechanisms are integral to protein homeostasis. Their reduced efficiency during aging leads to accumulation of misfolded and aggregated proteins which potentiate proteotoxic disorders. Paradoxically, our lab reported that the Caenorhabditis elegans rpn-10(ok1865) proteasome mutant possesses enhanced proteostasis and extended lifespan. RPN-10/PSMD4 is a ubiquitin receptor of the 26S proteasome that targets polyubiquitinated substrates to its catalytic core for degradation. Proteasome dysfunction of the rpn-10 mutant is characterized by reduced, not inhibited, ubiquitin fusion degradation. We ascertained that upregulated autophagy and SKN-1/Nrf-mediated responses partially contribute to the robust rpn-10 mutant phenotype. Further investigation of its underlying mechanism revealed that several ERQC genes are transcriptionally upregulated in the rpn-10 mutant. Thus, we hypothesized that the rpn-10 mutant exhibits improved ER proteostasis which mediates its elevated cellular stress resistance. Accordingly, the rpn-10 mutant shows increased ER stress resistance and altered ER homeostasis. Complementarily, attenuated expression of the aggregation-prone α-1 antitrypsin (ATZ) reporter proves that ER proteostasis is ameliorated in the rpn-10 mutant. Via a genetic screen for suppressors of decreased ATZ aggregation in the rpn-10 mutant, we identified novel player H04D03.3, which is a homolog of the proteasome adaptor ECM29. This suggests that assembly of the rpn-10 mutant proteasome itself critically regulates its ER proteostasis. Moreover, we observed that cytosolic proteostasis and longevity depend on ER master chaperone hsp-3/-4(BiP) and ER ATPase cdc-48.2(p97/VCP), further highlighting ERQC significance in the rpn-10 mutant. Altogether, it appears that mild proteasomal dysfunction induces ERQC adaptation that underlies proteostasis and longevity benefits of the rpn-10 mutant.

